# Serum adipokines play different roles in type I and II ketosis

**DOI:** 10.5713/ajas.19.0728

**Published:** 2019-12-24

**Authors:** Liuhong Shen, Yingkun Zhu, Jinbang Xiao, Bolin Qian, Liuchao You, Yue Zhang, Shumin Yu, Xiaolan Zong, Suizhong Cao

**Affiliations:** 1 Department of Clinical Veterinary Medicine, Veterinary Medicine College, Sichuan Agricultural University, Chengdu 611130, China

**Keywords:** Adipokines, Insulin Resistance, Ketosis, Negative Energy Balance

## Abstract

**Objective:**

This study was conducted to investigate the differences in several serum adipokines in perinatal dairy cows with type I and II ketosis, and the correlations between these adipokines and the two types of ketosis.

**Methods:**

Serum adiponectin (ADP), leptin (LEP), resistin, tumor necrosis factor-α (TNF-α), and interleukin-6 (IL-6) levels, and energy balance indicators related to ketosis were measured. Type I and II ketosis were distinguished by serum glucose (Glu) and Y values and the correlations between adipokines in the two types of ketosis were analyzed.

**Results:**

β-Hydroxybutyric acid of type I ketosis cows was significantly negatively correlated with insulin (INS) and LEP and had a significant positive correlation with serum ADP. In type II ketosis cows, ADP and LEP were significantly negatively correlated, and INS and resistin were significantly positively correlated. Revised quantitative INS sensitivity check index (RQUICKI) values had a significantly positive correlation with ADP and had a very significant and significant negative correlation with resistin, TNF-α, and IL-6. ADP was significantly negatively correlated with resistin and TNF-α, LEP had a significantly positive correlation with TNF-α, and a significantly positive correlation was shown among resistin, IL-6, and TNF-α. There was also a significant positive correlation between IL-6 and TNF-α.

**Conclusion:**

INS, ADP, and LEP might exert biological influences to help the body recover from negative energy balance, whereas resistin, TNF-α, and IL-6 in type II ketosis cows exacerbated INS resistance and inhibited the production and secretion of ADP, weakened INS sensitivity, and liver protection function, and aggravated ketosis.

## INTRODUCTION

Ketosis is one of the main metabolic diseases that is caused by negative energy balance (NEB) and induces glucose (Glu) and lipid metabolic disorders in perinatal dairy cows. The incidence of ketosis in high-yielding cattle can be up to 30% [1] and can lead to lactation and milk quantity decline, a higher risk of secondary disease, and great economic losses [2]. Currently, a blood β-hydroxybutyric acid (BHBA) ≥1.2 mmol is generally used as the “gold standard” for the diagnosis of dairy cow ketosis [3], with ketosis divided into type I and type II. Type I is mainly caused by nutrition deficiency, occurs at 3 to 6 weeks postpartum, and shows high serum BHBA and non-esterified fatty acid (NEFA) concentrations with low serum Glu level. Type II ketosis is pathological and is based on the liver function disorder caused by a fatty liver. This ketosis type has characteristics of high blood BHBA (but lower than type I ketosis), NEFA, and insulin (INS) concentrations with hyperglycemia that shows insulin resistance (IR), and mainly occurs at 1 to 2 weeks postpartum [4].

Adipokines are cytokines that are secreted by adipocytes and mediate a series of signaling pathways by binding to adipokine receptors in various tissues and organs [5,6]. Adipokines are involved in the metabolic and endocrine regulations of the body, and primarily regulate energy balance, oxidative stress, and inflammatory response by affecting INS sensitivity and Glu–lipid metabolism, which have important regulating functions for human diabetes, obesity, and metabolic syndrome [7–9]. Many studies have been undertaken on adiponectin (ADP), leptin (LEP), resistin, and interleukin-6 (IL-6), which are closely related to energy metabolism, as well as tumor necrosis factor-α (TNF-α) [10]. However, no studies have investigated whether adipokines are also involved in metabolic regulation such as Glu metabolism and lipid mobilization in dairy cows and their relationship with ketosis. Therefore, the present study explored the correlation between partial serum adipokine concentrations and ketosis in cows. It also measured the regulation and interaction function of adipokines in ketosis cows, providing a new research method for the diagnosis and treatment of cow ketosis, and investigating the complex glycolipid metabolism network.

## MATERIALS AND METHODS

### Experimental animals

A total of 108 cows with ketosis, including 52 cows with type I ketosis and 56 cows with type II ketosis were selected by diagnosis from 412 included in the study. The postpartum (within 42 days after delivery) Holstein cows were 2 to 4 parity, weight of 578±42 kg, and body condition score of 3.29±0.35. They were maintained in a semi-enclosed area with unified feeding in a large-scale dairy farm in Sichuan Province. Based on the average lactation days that ketosis was discovered, 59 healthy cows with similar lactation days (±3 days) were selected as the control groups.

### Test equipment

The following equipment was used during the study: micro-oscillator (WZS-III, Jincheng Instrument Factory, Wuxi, China); centrifuge (model LD4-2A, Beijing Centrifuge Factory, Beijing, China); microplate reader (ELx800, BIO-TEK, Winooski, VT, USA); hot constant temperature water bath (HH-6 with digital display, Guohua Electric Co., Ltd., Suzhou, China); and spectrophotometer 722 (Dawei Instrument Equipment Co., Ltd, Nanjing, China).

### Test reagents

The following reagents were used during the study: bovine ADP (sensitivity 0.1 μg/mL), LEP (0.1 ng/mL), resistin (0.1 ng/mL), TNF-α (0.1) ng/L, IL-6 (0.01 pg/mL), INS (0.078 IU/mL) ELISA kit (accuracy>0.92), NEFA double reagent enzymatic detection kit, TG GRP-PAP enzymatic assay kit, Glu oxidase assay kit, BHBA Enzyme Colorimetric Test Kit, and an aspartate aminotransferase (AST) microplate kit. All reagents were purchased from Jiancheng Biotechnology Co., Ltd, Nanjing, China.

### Sample collection and processing

A 10 mL tail venous blood sample from the 412 experimental cows was collected at 06:00 h (within 42 days after delivery) and the sampling date was recorded. Collected venous blood was placed in a centrifuge tube without anticoagulant and centrifuged at 3,000 rpm for 10 min to separate the serum. After 1 h, the sample was kept at room temperature (25°C to 30°C) and the upper serum was transferred to an Eppendorf tube and stored at −20°C.

### Detection of energy balance indicators related to serum adipokines and ketosis

Serum ADP, LEP, resistin, TNF-α, IL-6, BHBA, NEFA, AST, triglyceride, Glu, and INS concentrations were tested by strictly following the manufacturer’s instructions of the kits.

### Diagnosis of type I and type II ketosis in dairy cows

Cows with serum BHBA concentrations >1.2 mmol/L were determined to have ketosis. According to the fatty liver diagnosis formula established by Reid et al [11], a fatty liver can be diagnosed by serum NEFA, Glu, and AST levels: Y = −0.51–0.003NEFA+2.84Glu–0.0528AST. The fatty liver is severe when Y<0, moderate when 0<Y<1, and there is no fatty liver when Y>1. To secure accuracy of the diagnoses, cows with Glu<2.5 mmol/L and Y>0 were considered to have type I ketosis, whereas cows with Glu>2.8 mmol/L and Y<0 were considered as type II ketosis in the present study [12].

### Data analysis and processing

The SPSS 24.0 software program was used to calculate the INS sensitivity index (revised quantitative insulin sensitivity check index [RQUICKI]) = 1/([l g Glu]+[l g NEFA]+[l g INS]). Correlations between serum ADP, LEP, resistin, TNF-α, and IL-6 concentrations, and serum BHBA, NEFA, and RQUICKI levels were also analyzed and their distribution tested with SPSS 24.0. Each data was expressed as &Xmacr;±S form and correlations were tested using Pearson’s correlation test and considered to be significant or very significant if p<0.05 or p<0.01, respectively.

## RESULTS

### Diagnosis of type I and type II ketosis

A total of 52 and 56 cows were diagnosed with type I and II ketosis, respectively, in which serum Glu, AST, and Y value levels of type I ketosis cows were very significantly lower than those of type II ketosis cows (Table 1).

### Analysis of serum parameter differences in type I and type II ketosis cows and healthy cows

The incidence day that type I ketosis was measured was very significantly later than that for type II ketosis, serum ADP concentrations and RQUICKI levels in the type I ketosis group were significantly higher than that in the type II ketosis group, and INS concentrations of both control groups were significantly higher than that of the type II ketosis group. The serum INS levels in the healthy and type I ketosis groups were significantly lower than that of the type II ketosis group, and serum BHBA concentration of the type I ketosis group was very significantly higher than that of the type II ketosis group (Table 2). Serum LEP, resistin, TNF-α, and IL-6 showed no significant differences between the two ketosis groups. The type II ketosis group showed a significantly higher TNF-α than that of control group II.

### Analysis of the correlation between partial adipokines and type I ketosis

Serum BHBA levels in type I ketosis cows had a very significant negative correlation with INS levels and LEP levels (r = −0.684 and −0.862, respectively); however, these levels were very significantly positively correlated with serum ADP levels (r = 0.697) (Table 3). Serum ADP concentration was very significantly negatively correlated with LEP (r = −0.862) and the correlations between the other serum markers were not significant (p>0.05) (Table 3).

### Analysis of the correlation between partial adipokines and type II ketosis

BHBA concentration had no significant correlations with any other indicators in type II ketosis cows (p>0.05) (Table 4). Serum INS concentrations had significantly positive and negative correlations with resistin (r = 0.541) and RQUICKI (r = −0.549) levels, respectively. Serum RQUICKI was significantly positively correlated with ADP concentrations (r = 0.591) and had very significant negative correlations with resistin and TNF-α levels (r = −0.757 and −0.721, respectively), and a significant negative correlation with IL-6 levels (r = −0.533). Serum ADP levels were significantly negatively correlated with resistin and TNF-α (r = −0.581 and −0.596, respectively). LEP concentration had a significantly positive correlation with TNF-α (r = 0.653); the serum resistin level was significantly positively correlated with IL-6 and TNF-α (r = 0.653 and 0.589, respectively); and there was a significant positive correlation between IL-6 and TNF-α (r = 0.762).

## DISCUSSION

### Partial energy balance and adipokines index of type I and type II ketosis cows

Ketosis in dairy cows can be divided into type I and type II ketosis based on differences in pathology and blood index. Type I ketosis, also known as hunger-type ketosis, is mainly induced by insufficient raw sugar occurring within six weeks after delivery and is characterized by high serum ketone and NEFA concentrations, and hypoglycemia [13,14]. The key to the prevention and treatment of type I ketosis is restoring feed intake and supplementing raw sugar. Type II ketosis in dairy cows is similar to type II diabetes in humans, with a pathologic basis of a fatty liver and IR [12,15,16]. Type II ketosis mainly occurs within 1 to 2 weeks postpartum, is induced by lipid deposition and gluconeogenesis reduction, and has high serum ketone, NEFA, and INS concentrations and slight hyperglycemia (or average Glu level). This type of ketosis can be prevented by perinatal weight control [17]. Xu et al [4] found differences in blood physicochemical parameters and physiological indexes between type I and type II ketosis. The blood BHBA and NEFA concentrations of type I ketosis cows in the present study were significantly higher than those of type II ketosis cows; however, the blood sugar levels and body condition scores were significantly lower. INS was also lower in type I than in type II ketosis cows; however, the difference was not significant. In the present study, the serum BHBA concentration of type I ketosis cows was significantly higher than that of type II ketosis cows, which was consistent with the results of Xu et al [4]. Therefore, NEFA oxidation and lipid mobilization are better when liver function is normal. The serum INS level of type II ketosis cows was significantly higher than that of type I ketosis cows; however, the RQUICKI was significantly lower, confirming that cows with type II ketosis were associated with severe IR.

ADP is the only protective adipokine that inhibits lipogenesis and glycogen output and activates fatty acid oxidation, thereby reducing fat deposition in the liver. ADP also acts as a sensitizer for INS and a decrease in its level leads to a decline in intracellular INS receptor activity, which induces IR. Siddiqui et al [18] stated that the potential mediators of IR (such as ADP, LEP, and resistin) are involved in tissue INS secretion and the regulation of INS sensitivity directly, and they affect Glu and lipid metabolism. Our results showed that serum ADP levels in type II ketosis cows were significantly lower than that in type I ketosis cows, indicating that the ADP level was potentially associated with type II ketosis by ADP decline induced by the fatty liver and IR. LEP has a two-way regulation effect on INS, i.e., high LEP concentration can promote fat decomposition into NEFA, which interferes with the sensitivity of INS in the muscle, inducing IR and causing fatty liver and type II ketosis under pathological conditions.

In the present study, the serum LEP concentration in type II ketosis cows was higher than that in type I ketosis cows; however, the difference was not significant, which may be due to the high body fat and obesity of dairy cows, and resistin being associated with obesity and IR, which is produced by islets and regulates the secretion of INS and glucagon *in vitro* [19]. The ADP, LEP, and resistin are involved in INS secretion in tissues and regulate INS sensitivity directly while also being involved in the inflammatory response, affecting the accumulation of adipose tissue and influencing Glu metabolism [18]. In the present study, the resistin in type II ketosis cows was higher than that in type I ketosis cows; however, the difference was not significant, which may be related to the induction of IR. Both IL-6 and TNF-α play essential roles in the development of IR and previous studies have found that TNF-α and IL-6 are involved in the occurrence and development of the nonalcoholic fatty liver disease, and are important in clinical diagnosis in humans, thus contributing to the diagnosis and treatment of the disease [16]. In the present study, serum TNF-α and IL-6 in type II ketosis cows were higher than in type I ketosis cows but not significantly, indicating a fatty liver in type II ketosis cows may be associated with IR. No significance is probably caused by postpartum lipid mobilization and open cervix leading to the secretion of inflammatory adipokines and immune or inflammatory response of endometrial mucosa [20].

### Correlation between partial adipokines and type I ketosis

**Figure 1:**
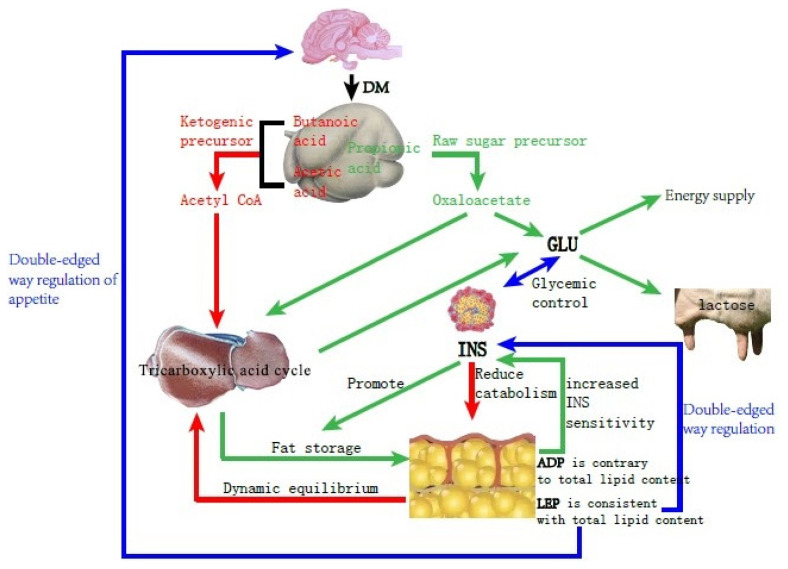
Schematic diagram of Glucose and lipid metabolism in dairy cows with energy balance.

**Figure 2:**
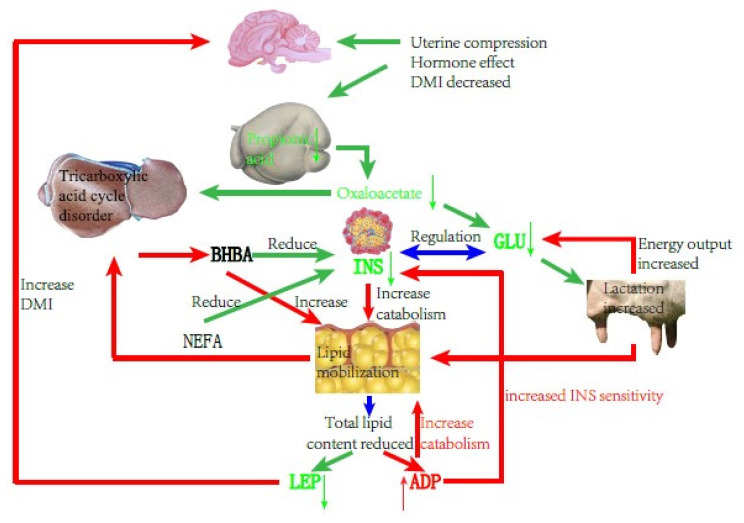
Schematic diagram of cows with type I ketosis.

Dairy cows consume vast amounts of energy during delivery and the demand for energy during the lactation period increases; however, the rumen of the cow is squeezed by the uterus at the end of pregnancy and the secretion of reproductive hormones inhibits appetite, resulting in decreased feed intake and slow recovery, making energy requirements exceed intake. Therefore, the normal metabolic pathway (Figure 1) changes and the NEFA produced by fat mobilization is incompletely oxidized to produce acetoacetate and BHBA, causing BHBA to accumulate (Figure 2), and type I cows usually have hypoglycemia, low INS, high BHBA, and high NEFA [21].

INS is one of the critical hormones regulating sugar and lipid metabolism. In type I ketosis, INS is affected by NEB, high NEFA, and high BHBA. On the one hand, type I ketosis cows are in the hypoglycemia situation because of their high energy requirements and insufficient feed intake, and blood sugar stimulation to islet B cell is relieved, thus INS secretion is inhibited [13]. On the other hand, a low level of INS promotes fat mobilization, and long-term high NEFA and BHBA concentrations inhibits Glu metabolism in islet cells and causes islet cell apoptosis, leading to progressive body fat decomposition and exacerbating ketosis. In our experiment, the serum INS and BHBA concentrations of type I ketosis cows showed a very significant negative correlation. Li et al [13] reported that the serum INS of type I ketosis cows was similar to that of normal cows, indicating that the INS level was mainly affected by systemic fat mobilization and negative feedback of blood Glu level in type I ketosis. This reduces the secretion of INS and the synthesis of glycogen, which benefits fat mobilization; however, it is still within reasonable biological regulation. BHBA reflects the severity of ketosis, with long-term low INS levels aggravating fat mobilization that increases BHBA and deteriorates ketosis, whereas high NEFA and BHBA levels inhibit INS secretion and INS sensitivity in the liver and adipose tissue, establishing a vicious cycle. ADP is the only factor that is secreted by white adipose tissue and has a negative correlation with fat capacity and obesity [22]. As an INS sensitizer, ADP can enhance INS sensitivity in a variety of ways. In the present study, there was no significant correlation between ADP and INS, probably due to the complex changes in the glycolipid metabolism network in NEB cows, and ADP and INS were regulated by various other factors. ADP can regulate lipid metabolism by promoting the oxidation of fatty acids [23–26] and inhibiting the synthesis of lipids [24,27] and finally results in the lowering of lipids. In the present study, serum ADP and BHBA concentrations of type I ketosis cows were significantly positively correlated. To meet the required energy demand, type I ketosis cows mobilize body fat and the increase of BHBA shows an increase in the degree of fat decomposition. ADP plays a biological role in promoting fat decomposition, which is conducive to the recovery of NEB status in dairy cows, with these results being similar to those of Yu et al [28].

LEP is mainly involved in the regulation of food intake, INS sensitivity, and weight maintenance, and is also a “messenger” that reflects the fat content of the body. LEP can activate the kinase and transcriptional activator pathways for signal transduction, and affects a variety of neuroendocrine hormones such as neuropeptides and melanocytes, causing appetite changes. Previous studies have shown that LEP can inhibit the binding of INS to its receptor and controls the production of INS *in vivo*. A high concentration of LEP can inhibit INS and vice versa. In the present study, the serum LEP of type I ketosis cows was significantly negatively correlated with BHBA. Postpartum fat mobilization in dairy cows occurred before BHBA accumulation and the decrease in whole body fat content led to a decrease in LEP secretion. After the type I ketosis, a large amount of BHBA caused an increase in fat breakdown and the body fat reserve was further reduced, resulting in a decrease in serum LEP content. Decreased serum LEP stimulates the hypothalamus to increase the excitability of the parasympathetic nerves, helps the cows to resume feeding, reduces body fat decomposition, and promotes the production of INS. It is speculated that LEP may play a potential mitigation role in type I ketosis.

In summary, type I ketosis occurs due to insufficient intake of raw food and sugar, and a large amount of mobilization of body fat beyond the metabolic capacity of the body. NEB, because of increased nutrition requirement and appetite decline during the postpartum period, affects serum INS, ADP, and LEP concentrations. Thus, serum INS, ADP, and LEP can reflect energy metabolism conditions in the body and retain normal regulation functions such as lipid decomposition and increasing appetite, helping with relief from NEB.

### Correlation between partial adipokines and type II ketosis

IR and fatty liver are important pathologic bases of type II ketosis in dairy cows, and Xu et al [15] confirmed that there was also IR in ketosis and NEB cows. Adipokines can participate in IR by regulating and affecting INS sensitivity and interaction, suggesting that they mediate fatty liver and type II ketosis in dairy cows. Among them, ADP increases the sensitivity of INS in the liver and skeletal muscle by activating Adenosine 5′-monophosphate activated protein kinase and peroxisome proliferators-activated receptors-α signaling pathways, thereby increasing the body’s consumption of fatty acids and energy, increasing the level of fatty acid transporter mRNA in the muscle, and accelerating the clearance of plasma NEFA. However, ADP can antagonize TNF-α, inhibit its secretion, and promote the role of IR [29]. High levels of LEP will promote fat decomposition, increase the production of NEFA, and interfere in the INS sensitivity of muscle tissue. This causes a decline in INS inactivation in the liver, leading to IR and liver steatosis. Resistin is an adipokine that induces IR, and reduces the sensitivity of various tissues to INS and the ability to take up glycogen, thus causing and aggravating IR through interactions in the liver, adipose tissue, skeletal muscle, and other hormones and enzymes. IL-6 and TNF-α are non-specifically secreting inflammatory factors of adipose tissue and can cause, meditate, or participate in inflammation, inhibiting INS sensitivity and triggering IR [18,30]. TNF-α can inhibit ADP production and compete with ADP for the same receptor using a similar structure. TNF-α can stimulate the production of IL-6 and the two produce nitric oxide directly or together, which acts on islet B cells to induce apoptosis [31,32]. IL-6 can inhibit ADP expression and competes with the LEP receptor in the same signaling pathway. High IL-6 levels can inhibit LEP binding to the LEP receptor and trigger LEP resistance, which occurs with or exacerbates IR [33].

In the present study, there was no significant correlation between serum BHBA and other indicators in the type II ketosis cows, but it was correlated with LEP, resistin, IL-6, and TNF-α, indicating that IR may cause the pathologic basis and development of type II ketosis. Thus, high levels of LEP, resistin, and inflammatory-related adipokines capable of causing IR may promote the occurrence and development of type II ketosis. RQUICKI reflects the sensitivity of the human body to INS and is equally applicable to dairy cows [34]. In the present study, RQUICKI was significantly positively correlated with ADP, but not with resistin, TNF-α, and IL-6. There was a very significant negative correlation with resistin and a significant negative correlation with TNF-α and IL-6; therefore, ADP played a role in enhancing INS sensitivity, but resistin, TNF-α, and IL-6 reduced INS sensitivity and promoted IR. These results are similar to those of Wei et al [35] from human nonalcoholic fatty liver disease. There was a significant negative correlation between ADP and resistin and TNF-α, indicating that ADP and resistin have opposite biological functions, and when TNF-α levels were high, ADP production was inhibited and the sensitization effect on INS was decreased. LEP was significantly positively correlated with TNF-α, indicating that LEP levels were higher in type II ketosis cows than in type I ketosis cows, which may play a role in reducing INS sensitivity and exacerbating IR with other inflammatory adipokines under the pathologic basis of a fatty liver. There was a significantly positive correlation between resistin and IL-6 and TNF-α, and a very significantly positive correlation between IL-6 and TNF-α, indicating that among the type II ketosis cows, these three adipokines played a role in reducing INS sensitivity, blocked INS signal transduction together, and participated in IR. IL-6 and TNF-α are not only affected by inflammation, such as from a fatty liver, but they also have a promotional relationship with each other, which is consistent with the results of Wu et al [36].

**Figure 3:**
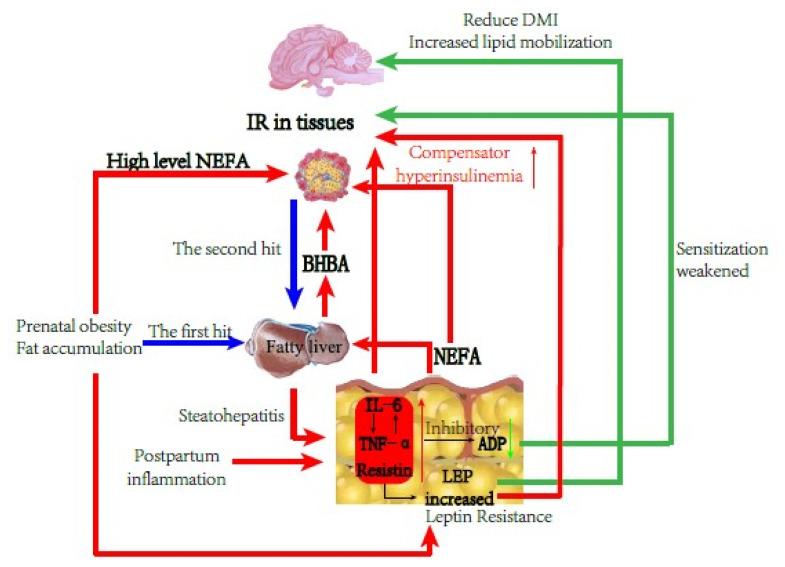
Schematic diagram of cows with type II ketosis.

In summary, type II ketosis occurs on the pathologic basis of IR and fatty liver. In the persistently slight inflammation situation induced by obesity and liver fat infiltration, the concentrations of inflammatory adipokines such as TNF-α and IL-6 increased and aggravated IR further, while simultaneously caused the inhibition of ADP production and secretion. Therefore, downregulated ADP levels decreased the INS sensitization and liver protection functions, interfered with Glu and lipid metabolism, and enhanced IR and liver fat deposition. Cows mobilize body fat after delivery, weakening liver NEFA oxidation function, and ketosis occurs within 1 to 2 weeks after birth, which further causes glycolipid metabolism disorder (Figure 3).

There are differences in the blood energy balance index and partial adipokines between type I and type II ketosis cows, suggesting a difference in the pathogenesis of the two types of ketosis. The changes of serum adipokines in type I ketosis cows were more affected by NEB, but still promoted fat decomposition and increased appetite to help cows alleviate NEB. The levels of resistin, TNF-α, and IL-6 in type II ketosis cows aggravated the pathologic basis of IR, while inhibiting the production and secretion of ADP, leading to reduced INS sensitization and liver protection, increased IR, and liver lipid deposition that increase the risk of ketosis.

## Figures and Tables

**Table 1:** Indicators relative to grouping

Index	Type I ketosis cows (n = 52)	Type II ketosis cows (n = 56)
Glu (mmol/L)	2.08±0.36^B^	2.98±0.45^A^
NEFA (mmol/L)	1.33±0.33^a^	1.15±0.37^a^
AST (U/L)	88.06±17.23^B^	155.43±24.12^A^
Y1)	0.74±0.24^A^	−0.26±0.31^B^

NEFA, non-esterified fatty acid; AST, aminotransferase;

1) Y = −0.51–0.003NEFA+2.84Glu–0.0528AST.

a,b There are significant differences between different lower-case letters marked on the same line (p<0.05).

A,B There are highly significant differences between different upper-case letters marked on the same column (p<0.01).

**Table 2:** The blood index of type I, II ketosis cows and healthy cows

Blood index	Type I ketosis cows (n = 52)	Control group I (n = 28)	Type II ketosis cows (n = 56)	Control group II (n = 31)
Incidence day (postpartum)	23.22±11.18^A^	22.86±2.10^A^	12.10±5.45^B^	12.10±2.09^B^
BHBA (mmol/L)	2.21±0.70^A^	0.79±0.29^C^	1.58±0.35^B^	0.71±0.25^C^
INS (μIU/mL)	4.94±0.51^B^	5.13±0.83^B^	5.84±0.61^A^	4.85±0.87^B^
RQUICKI	0.90±0.07^a^	0.96±0.14^a^	0.80±0.26^b^	0.92±0.16^a^
ADP (mg/L)	37.61±4.98^a^	35.32±4.01^ab^	32.67±6.07^b^	36.57±5.59^ab^
LEP (ng/mL)	2.53±0.88^a^	2.79±1.04^a^	2.73±0.84^a^	2.55±0.60^a^
Resistin (ng/mL)	54.51±7.86^a^	45.17±13.13^b^	61.66±12.67^a^	49.93±15.08^b^
IL-6 (ng/L)	244.53±49.17^a^	224.24±51.25^b^	275.63±29.87^a^	230.71±52.46^b^
TNF-α (ng/L)	140.92±16.27^ab^	129.89±30.93^ab^	145.54±16.92^a^	125.23±31.98^b^

BHBA, β-hydroxybutyric acid; INS, insulin; RQUICKI, revised quantitative insulin sensitivity check index; ADP, adiponectin; LEP, leptin; IL-6, interleukin-6; TNF-α, tumor necrosis factor-α.

a,b There are significant differences between different lower-case letters marked on the same line (p<0.05).

A–C There are highly significant differences between different upper-case letters marked on the same column (p<0.01).

**Table 3:** The correlation analysis between adipokines and type I ketosis

Index	BHBA	INS	RQUICKI	ADP	LEP	Resistin	IL-6	TNF-α
BHBA	-	−0.684**	−0.120	0.697**	−0.862**	0.052	0.336	0.215
INS	-	-	−0.476	−0.460	0.385	0.272	0.09	−0.016
RQUICKI	-	-	-	0.335	0.256	0.017	−0.244	−0.003
ADP	-	-	-	-	−0.638*	0.022	0.081	−0.456
LEP	-	-	-	-	-	0.079	−0.349	0.279
Resistin	-	-	-	-	-	-	0.219	0.946
IL-6	-	-	-	-	-	-	-	0.484
TNF-α	-	-	-	-	-	-	-	-

BHBA, β-hydroxybutyric acid; INS, insulin; RQUICKI, revised quantitative insulin sensitivity check index; ADP, adiponectin; LEP, leptin; IL-6, interleukin-6; TNF-α, tumor necrosis factor-α.

* Indicates significant difference (p<0.05),

** indicates extremely significant difference (p<0.01).

**Table 4:** The correlation analysis between adipokines and type II ketosis

Index	BHBA	INS	RQUICKI	ADP	LEP	Resistin	IL-6	TNF-α
BHBA	-	0.303	−0.428	−0.016	0.425	0.479	0.452	0.366
INS	-	-	−0.549*	−0.411	0.407	0.541*	0.104	0.199
RQUICKI	-	-	-	0.591*	−0.368	−0.757**	−0.533*	−0.721**
ADP	-	-	-	-	−0.022	−0.581*	−0.437	−0.596*
LEP	-	-	-	-	-	0.439	0.503	0.653*
Resistin	-	-	-	-	-	-	0.653*	0.589*
IL-6	-	-	-	-	-	-	-	0.762**
TNF-α	-	-	-	-	-	-	-	-

BHBA, β-hydroxybutyric acid; INS, insulin; RQUICKI, revised quantitative insulin sensitivity check index; ADP, adiponectin; LEP, leptin; IL-6, interleukin-6; TNF-α, tumor necrosis factor-α.

* Indicates significant difference (p<0.05),

** indicates extremely significant difference (p<0.01).

## References

[1] Ruprechter G, Adrien ML, Larriestra A (2018). Metabolic predictors of peri-partum diseases and their association with parity in dairy cows. Res Vet Sci.

[2] Sheldon IM, Cronin J, Goetze L, Donofrio G, Schuberth HJ (2009). Defining postpartum uterine disease and the mechanisms of infection and immunity in the female reproductive tract in cattle. Biol Reprod.

[3] Tatone EH, Duffield TF, LeBlanc SJ, DeVries TJ, Gordon JL (2017). Investigating the within-herd prevalence and risk factors for ketosis in dairy cattle in Ontario as diagnosed by the test-day concentration of beta-hydroxybutyrate in milk. J Dairy Sci.

[4] Xu C, Li Y, Xia C, Zhang H, Sun L, Xu C (2015). ^1^H NMR-based plasma metabolic profiling of dairy cows with type I and type II ketosis. Pharm Anal Acta.

[5] Samad F, Badeanlou L, Shah C, Yang G, Cowart LA (2011). Adipose tissue and ceramide biosynthesis in the pathogenesis of obesity. Sphingolipids and metabolic disease. Advances in Experimental Medicine and Biology.

[6] Luo M, Guo J, Huang G, Lu B, Yu W (2014). Advances in research on the relationship between adipokines and metabolic syndrome. Progress in Modern Biomedicine.

[7] Wang Y, Xu A, Knight C, Xu LY, Cooper GJS (2002). Hydroxylation and glycosylation of the four conserved lysine residues in the collagenous domain of adiponectin. Potential role in the modulation of its insulin-sensitizing activity. J Biol Chem.

[8] Ingvartsen KL, Boisclair YR (2001). Leptin and the regulation of food intake, energy homeostasis and immunity with special focus on periparturient ruminants. Domest Anim Endocrin.

[9] Deng Y, Scherer PE (2010). Adipokines as novel biomarkers and regulators of the metabolic syndrome. Ann NY Acad Sci.

[10] Kumari R, Kumar S, Kant R (2019). An update on metabolic syndrome: Metabolic risk markers and adipokines in the development of metabolic syndrome. Diabetes Metab Syndr Clin Res Rev.

[11] Reid IM, Dew SM, Collins RA, Ducker MJ, Bloomfield GA, Morant SV (1983). The relationship between fatty liver and fertility in dairy cows: a farm investigation. J Agric Sci.

[12] Wang J (2013). Comparison and analysis of blood metabolic profiles between healthy cows with ketosis and subclinical hypocalcemia in perinatal period [dissertation].

[13] Li Y, Xu C, Xia C, Zhang H, Sun L, Gao Y (2014). Plasma metabolic profiling of dairy cows affected with clinical ketosis using LC/MS technology. Vet Q.

[14] Zhang B, Guo H, Yang W (2019). Effects of ORAI calcium release-activated calcium modulator 1 (ORAI1) on neutrophil activity in dairy cows with subclinical hypocalcemia. J Anim Sci.

[15] Xu C, Shu S, Xia C, Wang B, Zhang HY, Jun B (2014). Investigation on the relationship of insulin resistance and ketosis in dairy cows. J Vet Sci Technol.

[16] Cui Y, Wang Q, Chang R, Zhou X, Xu C (2019). Intestinal barrier function–non-alcoholic fatty liver disease interactions and possible role of gut microbiota. J Agric Food Chem.

[17] Li X, Li Y, Ding H (2018). Insulin suppresses the AMPK signaling pathway to regulate lipid metabolism in primary cultured hepatocytes of dairy cows. J Dairy Res.

[18] Siddiqui K, George TP (2017). Resistin role in development of gestational diabetes mellitus. Biomark Med.

[19] Sassek M, Pruszynska-Oszmalek E, Kolodziejski PA (2016). Resistin is produced by rat pancreatic islets and regulates insulin and glucagon *in vitro* secretion. Islets.

[20] Cha S, Li Y, Zhang S, Feng P (2016). Detection and analysis of plasma TNF-α and IL-6 in cows with endometritis. Chinese Cow.

[21] Budras KD, Habel RE, Mülling KW, Greenough PR (2011). Bovine anatomy.

[22] Cao T (2014). Investigation on the protective action of adiponectin against atrial fibrillation [dissertation].

[23] Mao X, Kikani CK, Riojas RA (2006). APPL1 binds to adiponectin receptors and mediates adiponectin signalling and function. Nat Cell Biol.

[24] Shen Z, Liang X, Rogers CQ, Rideout D, You M (2010). Involvement of adiponectin-SIRT1-AMPK signaling in the protective action of rosiglitazone against alcoholic fatty liver in mice. Am J Physiol Gastrointest Liver Physiol.

[25] Yoon MJ, Lee GY, Chung JJ, Ahn YH, Hong SH, Kim JB (2006). Adiponectin increases fatty acid oxidation in skeletal muscle cells by sequential activation of AMP-activated protein kinase, p38 mitogen-activated protein kinase, and peroxisome proliferator-activated receptor alpha. Diabetes.

[26] Heiker JT, Wottawah CM, Juhl C, Kosel D, Morl K, Beck-Sickinger AG (2009). Protein kinase CK2 interacts with adiponectin receptor 1 and participates in adiponectin signaling. Cell Signal.

[27] Awazawa M, Ueki K, Inabe K (2009). Adiponectin suppresses hepatic SREBP1c expression in an AdipoR1/LKB1/AMPK dependent pathway. Biochem Biophys Res Commun.

[28] Yu G (2011). Effect of bovine recombinant adiponectin on key enzymes of fat metabolism in dairy cows [dissertation].

[29] Yamauchi T, Kamon J, Waki H (2001). The fat-derived hormone adiponectin reverses insulin resistance associated with both lipoatrophy and obesity. Nat Med.

[30] Yu-Duan T, Chao-Ping W, Chih-Yu C (2013). Elevated plasma level of visfatin/pre-b cell colony-enhancing factor in male oral squamous cell carcinoma patients. Med Oral Patol Oral Cir Bucal.

[31] Huang S, Xu Y, Peng WF (2018). Asymmetric dimethylarginine targets MAPK pathway to regulate insulin resistance in liver by activating inflammation factors. J Cell Biochem.

[32] Wang Y, He B, Li X (2006). Changes and significance of TNF-α, IL-6 and NO in plasma of patients with hypertensive insulin resistance. J China Med Univ.

[33] Dali-Youcef N, Mecili M, Ricci R, Andres E (2013). Metabolic inflammation: connecting obesity and insulin resistance. Ann Med.

[34] Holtenius P, Holtenius K (2007). A model to estimate insulin sensitivity in dairy cows. Acta Vet Scand.

[35] Wei D, Zhao H, Ceng R, Zhang B (2018). Effect of phlegm and blood stasis therapy on inflammatory factors IL-6, IL-18 and TNF-α in nonalcoholic fatty liver disease. Chinese J Tradit Chinese Med.

[36] Wu Q, Jin Y, Ni H (2015). Effect of kaempferol on correlation factors of chronic complications of type 2 diabetic rats. Chinese Herbal and Herbal Drugs.

